# 

**Published:** 1874-03

**Authors:** 


### CONTENTS.

### COMMUNICATIONS.

Opening of the Pulp Cavity and Preparation of the Roots for Filling .......... 97
Contour Filling .......... 104
Lime Phosphates .......... 108
The Microscope .......... 112
Associated Effort .......... 114

### PROCEEDINGS OF SOCIETIES.

American Dental Society of Europe .......... 117
Discussions .......... 126

### SELECTIONS.

How to Deprive Iodine of its Stain .......... 131
The Proper Use of Cholagogues .......... 134

### EDITORIAL.

Screws .......... 137
The Morrison Engine Infringement .......... 138
History of Dentistry .......... 139
Ohio Dental College—Commencement .......... 140

